# Supplementary material for: Whole-brain tissue mapping toolkit using large-scale highly multiplexed immunofluorescence imaging and deep neural networks
Source: Nat Commun. 2021 Mar 10;12:1550. doi: 10.1038/s41467-021-21735-x (PMC7946933; doi:10.1038/s41467-021-21735-x)
Supplement: Supplementary file 3 — Description of Additional Supplementary Files [file 41467_2021_21735_MOESM3_ESM.pdf]

Title: Supplementary data 1
Description: Cell phenotyping results.
