# Supplementary material for: Whole-brain tissue mapping toolkit using large-scale highly multiplexed immunofluorescence imaging and deep neural networks
Source: Nat Commun. 2021 Mar 10;12:1550. doi: 10.1038/s41467-021-21735-x (PMC7946933; doi:10.1038/s41467-021-21735-x)
Supplement: Supplementary file 5 — Reporting Summary [file 41467_2021_21735_MOESM5_ESM.pdf]

nature research

Corresponding author(s): Badrinath Roysam, Dragan Maric

Last updated by author(s): Jan 15, 2020

Reporting Summary

Nature Research wishes to improve the reproducibility of the work that we publish. This form provides structure for consistency and transparency in reporting. For further information on Nature Research policies, see our Editorial Policies and the Editorial Policy Checklist.

Statistics

For all statistical analyses, confirm that the following items are present in the figure legend, table legend, main text, or Methods section.

n/a	Confirmed	
☐	☒	The exact sample size (n) for each experimental group/condition, given as a discrete number and unit of measurement
☐	☒	A statement on whether measurements were taken from distinct samples or whether the same sample was measured repeatedly
☒	☐	The statistical test(s) used AND whether they are one- or two-sided<br>*Only common tests should be described solely by name; describe more complex techniques in the Methods section.*
☒	☐	A description of all covariates tested
☐	☒	A description of any assumptions or corrections, such as tests of normality and adjustment for multiple comparisons
☐	☒	A full description of the statistical parameters including central tendency (e.g. means) or other basic estimates (e.g. regression coefficient) AND variation (e.g. standard deviation) or associated estimates of uncertainty (e.g. confidence intervals)
☒	☐	For null hypothesis testing, the test statistic (e.g. F, t, r) with confidence intervals, effect sizes, degrees of freedom and P value noted<br>*Give P values as exact values whenever suitable.*
☒	☐	For Bayesian analysis, information on the choice of priors and Markov chain Monte Carlo settings
☒	☐	For hierarchical and complex designs, identification of the appropriate level for tests and full reporting of outcomes
☒	☐	Estimates of effect sizes (e.g. Cohen's d, Pearson's r), indicating how they were calculated

Our web collection on statistics for biologists contains articles on many of the points above.

Software and code

Policy information about availability of computer code

Data collection	We used commercially available Zeiss ZEN 2 software for image acquisition.
Data analysis	We used Python 3.6, Matlab 2018b programming languages and FCSExpress v6.06 for data analysis. GitHub link for the source code is available at: https://github.com/RoysamLab/whole_brain_analysis/

For manuscripts utilizing custom algorithms or software that are central to the research but not yet described in published literature, software must be made available to editors and reviewers. We strongly encourage code deposition in a community repository (e.g. GitHub). See the Nature Research guidelines for submitting code & software for further information.

Data

Policy information about availability of data

All manuscripts must include a data availability statement. This statement should provide the following information, where applicable:

- Accession codes, unique identifiers, or web links for publicly available datasets
- A list of figures that have associated raw data
- A description of any restrictions on data availability

The datasets including the original acquired images from the microscope and the results of each step are publicly hosted at: https://www.dropbox.com/sh/yq9pnzwho9t4hyg/AABWUdsP_lWMGZrA0dqgoZ_Ka?dl=0
The cell nuclei detection model was trained on top of pre-trained faster_rcnn_inception_resnet_v2_atrous_coco model on ImageNet dataset. The pre-trained weights can be found on: https://github.com/tensorflow/models/blob/master/research/object_detection/g3doc/tf1_detection_zoo.md

Field-specific reporting

Please select the one below that is the best fit for your research. If you are not sure, read the appropriate sections before making your selection.

☒ Life sciences ☐ Behavioural & social sciences ☐ Ecological, evolutionary & environmental sciences

For a reference copy of the document with all sections, see nature.com/documents/nr-reporting-summary-flat.pdf

Life sciences study design

All studies must disclose on these points even when the disclosure is negative.

Sample size	The data consists of 50-channel stitched images scanned from 10-micron thick serial whole rat brain coronal tissue sections with the size of each image for each acquired channel approximately 2.5GB. The quantification analysis on individual sections resulted in detection of approximately 200,000 cells with 28-plex phenotypic information. We considered the provided sample size to be adequate for the purpose of demonstrating the effectiveness of our imaging and methods across extended brain regions.
Data exclusions	We did not exclude any data from the analyses.
Replication	From the quantitative analysis point, both the data and codes are made publicly available to anyone for reproduction.
Randomization	N/A because we are not comparing two or more populations.
Blinding	N/A because we are not comparing two or more populations

Reporting for specific materials, systems and methods

We require information from authors about some types of materials, experimental systems and methods used in many studies. Here, indicate whether each material, system or method listed is relevant to your study. If you are not sure if a list item applies to your research, read the appropriate section before selecting a response.

Materials & experimental systems

n/a	Involved in the study	
☐	☒	Antibodies
☒	☐	Eukaryotic cell lines
☒	☐	Palaeontology and archaeology
☐	☒	Animals and other organisms
☒	☐	Human research participants
☒	☐	Clinical data
☒	☐	Dual use research of concern

Methods

n/a	Involved in the study	
☒	☐	ChIP-seq
☒	☐	Flow cytometry
☒	☐	MRI-based neuroimaging

Antibodies

Antibodies used	All relevant antibody information has been provided in supplementary tables S1, S2, S5 and S6.
Validation	All antibodies used in this study have been previously validated in the literature and the validation information for each antibody is available from the source vendors where the antibodies were purchased. The relevant vendor information for each antibody is provided in supplementary tables S1, S2, S5 and S6.

Animals and other organisms

Policy information about studies involving animals; ARRIVE guidelines recommended for reporting animal research

Laboratory animals	8-week-old male Lewis LEW-Tg(CAG-EGFP)YsRrrc transgenic rats
Wild animals	No wild animals were used in the study.
Field-collected samples	No field collected samples were used in the study.
Ethics oversight	All animal handling procedures were approved by the Institutional Animal Care and Use Committee (IACUC) of the National Institute of Neurological Disorders and Stroke (NINDS, Bethesda, MD).

Note that full information on the approval of the study protocol must also be provided in the manuscript.
